# Accurate Tumor Segmentation via Octave Convolution Neural Network

**DOI:** 10.3389/fmed.2021.653913

**Published:** 2021-05-19

**Authors:** Bo Wang, Jingyi Yang, Jingyang Ai, Nana Luo, Lihua An, Haixia Feng, Bo Yang, Zheng You

**Affiliations:** ^1^The State Key Laboratory of Precision Measurement Technology and Instruments, Department of Precision Instrument, Tsinghua University, Beijing, China; ^2^Innovation Center for Future Chips, Tsinghua University, Beijing, China; ^3^Beijing Jingzhen Medical Technology Ltd., Beijing, China; ^4^School of Artificial Intelligence, Xidian University, Xi'an, China; ^5^Affiliated Hospital of Jining Medical University, Jining, China; ^6^China Institute of Marine Technology & Economy, Beijing, China

**Keywords:** liver, liver tumor, deep learning, octave convolution, segmentation

## Abstract

Three-dimensional (3D) liver tumor segmentation from Computed Tomography (CT) images is a prerequisite for computer-aided diagnosis, treatment planning, and monitoring of liver cancer. Despite many years of research, 3D liver tumor segmentation remains a challenging task. In this paper, we propose an effective and efficient method for tumor segmentation in liver CT images using encoder-decoder based octave convolution networks. Compared with other convolution networks utilizing standard convolution for feature extraction, the proposed method utilizes octave convolutions for learning multiple-spatial-frequency features, thus can better capture tumors with varying sizes and shapes. The proposed network takes advantage of a fully convolutional architecture which performs efficient end-to-end learning and inference. More importantly, we introduce a deep supervision mechanism during the learning process to combat potential optimization difficulties, and thus the model can acquire a much faster convergence rate and more powerful discrimination capability. Finally, we integrate octave convolutions into the encoder-decoder architecture of UNet, which can generate high resolution tumor segmentation in one single forward feeding without post-processing steps. Both architectures are trained on a subset of the LiTS (Liver Tumor Segmentation) Challenge. The proposed approach is shown to significantly outperform other networks in terms of various accuracy measures and processing speed.

## 1. Introduction

According to the World Health Organization, liver cancer was the second most common cause of cancer deaths in 2015. Hepatocellular carcinoma (HCC) is the most common primary liver cancer and the sixth most common cancer. Each year, the incidence and death rates of liver cancer are steadily increasing. In addition, the liver is also a common site for secondary tumors. It is an important factor leading to human death. With the rapid development of tumor radiation technology, radiotherapy has entered the stage of precision radiotherapy represented by image guidance and adaptive radiotherapy. Precision radiotherapy needs to accurately delineate the target area (tumor) of radiotherapy to guide treatment and subsequent radiation plans. But at this stage, accurate target area delineation in clinical medicine needs to be done manually by experienced physicians, and its accuracy and efficiency completely depend on the physician's clinical experience. This work is not only time-consuming, but also poorly reproducible.

Using computer image processing technology, combined with medical imaging diagnostic technology, early diagnosis, three-dimensional modeling, and quantitative analysis of liver diseases can enable doctors to have sufficient data before surgery, make preoperative planning, improve the success rate of surgery, and make reasonable preparations for an effective treatment plan. The accurate and reliable segmentation of liver contours from abdominal CT images is the first step in the early diagnosis of liver disease, the estimation of liver size and condition, and three-dimensional modeling. It is also a very critical step. The segmentation results have a direct effect on subsequent work. In actual clinical applications, the liver contour is usually manually segmented from CT images by physicians with relevant practical experience and professional knowledge. However, this process is very time-consuming and energy-consuming, and is subject to the subjective factors, experience, and knowledge of different physicians. The effect of the difference will often result in different segmentation results. Therefore, in order to reduce the workload of doctors, improve work efficiency, and obtain more objective and accurate segmentation results, computer-aided diagnosis technology must be introduced to help professional doctors segment liver CT images.

To solve this problem, researchers have invested in the research and come up with a number of approaches. Over the past few decades, they have focused on developing algorithms such as level sets, watershed, statistical shape models, region growth, active contour models, threshold processing, pattern cutting, and traditional machine learning methods that require manual extraction of tumor features.

Traditional liver segmentation methods are based on image processing methods, and mainly rely on some shallow features of the image, such as grayscale, statistical structure, and texture to segment liver contours. This feature can be obtained directly from the image or obtained by artificially designed extraction operators. These shallow features are less robust, not representative, and susceptible to noise interference. Practice has proved that it is often those abstract and deep features that are more representative. Deep learning technology can mine the deep abstract features of data from a large amount of data and apply them to liver segmentation tasks to improve the accuracy and robustness of segmentation.

Region growing, thresholding, or clustering methods have been widely used in medical image segmentation because they are fast, easy to implement, and have relatively low computational costs. However, the main drawback of these methods is that they use only strength information. As a result, this method is prone to boundary leakage at blurred tumor boundaries. Therefore, prior knowledge or other algorithms are integrated to reduce under-segmentation or over-segmentation (1–3). Anter et al. (1) present an automatic tumor segmentation method using adaptive region growth. A marker-controlled watershed algorithm was used to detect the initial seed points of regional growth. Yan et al. (4) present a semi-automatic segmentation method based on watershed transformation. They first manually placed seed points in the tumor area as markers, and then performed watershed transformation to delineate and extract tumor contours in the image. Therefore, the density information of the tumor can be obtained as a threshold to separate the hepatic lesion from its adjacent tissues. Then, the threshold is refined from the segmented lesion to obtain accurate results. DAS and Sabut (3) used adaptive thresholding, morphological processing, and nucleated fuzzy C-means (FCM) algorithms to segment liver tumors from CT images. Moghbel et al. (5) present an automatic tumor segmentation scheme based on supervised random Walker method. FCM with the function of cuckoo optimization is used for PIXEL marking of final random Walker segmentation.

Active contour methods, such as fast moving and level set algorithms, are popular segmentation techniques. However, good initialization and velocity function are needed to obtain accurate segmentation results, especially for tumors with uneven intensity and weak boundaries. Li et al. (6) present a new level set model that combines edge- and region-based information with prior information. An FCM algorithm is used to estimate the probability of tumor tissue. Li et al. (6) present a semi-automatic method for segmentation of liver tumors from magnetic resonance (MR) images, which uses a fast-moving algorithm to generate initial labeled regions and then classifies other unlabeled voxels through a neural network. A graph cutting method has also been widely used in medical image segmentation (7, 8), which can achieve global optimization solutions. Stawiaski et al. (7) present an interactive segmentation method based on watershed and graph cutting. When held in conjunction with the 2008 Liver Tumor Segmentation Challenge (LTSC08) competition [in conjunction with the 2008 Medical Image Computing and Computer-Assisted Intervention (MICCAI) conference], the method achieved the highest accuracy compared to other semi-automatic or automated methods. Linguraru et al. (8) present an automatic pattern segmentation method that uses pattern cutting with Hessian-based shape constraints to bias speckle-like tumors. However, the main drawback of such techniques based on level sets or graphic cuttings is their high computational cost, especially for 3D volume data.

Recently, deep learning (9–21) has penetrated into a variety of applications and surpassed the state-of-the-art performance in many fields such as image detection, classification, and segmentation (22–26), which also excites us to use this technique in the liver tumor segmentation task. Many researchers have already used deep learning methods to explore the task of liver tumor segmentation. In practical applications, CNN shows excellent feature extraction capabilities. Among them, fully convolutional neural networks (FCN) as an improved network of CNN have been widely used in the field of image segmentation. Different from image classification, semantic segmentation needs to determine the category of each pixel to achieve accurate segmentation. FCN replaces the last fully connected layer of CNN with a deconvolution layer to achieve pixel-to-pixel classification. The application of FCN and its derivatives in image segmentation continues to expand. Its encoder is the same as the 13 convolutional layers in VGG-16. The decoder maps the features extracted by the encoder to the encoder with the same resolution as the input. When the feature is extracted from small to small, the decoder gradually enlarges the extracted feature to the size of the input image from small to large. However, the traditional FCN network has poor edge segmentation and low accuracy, which cannot meet the requirements of medical image segmentation. Li et al. (6) propose a H-DensU-Net, which consists of 2D and 3D U-Net, for the segmentation of liver tumors. 2D U-Net is used to extract tumor features in individual sections, while 3D U-Net is used to understand tumor spatial information between sections. Sun et al. (27) present a method of liver tumor segmentation based on multi-channel full convolutional network (MC-FCN). They designed an MC-FCN to train contrast-enhanced CT images at different imaging stages, because each stage of the data provides unique information about the pathological features of the tumor. However, these neural networks are fully connected between adjacent layers, which leads to problems such as over-parameterization and over-fitting for tumor segmentation tasks. In addition, the number of trainable parameters in a fully connected neural network is related to the size of the input image, which results in higher computational costs when processing high-resolution images.

One of the challenges of deep learning for medical image processing is that the samples provided are often relatively small, and U-Net still performs well under this limitation. As an image semantic segmentation network, U-Net was mainly used to process medical images when it was proposed. The U-Net network is a CNN-based image segmentation network, mainly used for medical image segmentation. When it was first proposed, it was used for cell wall segmentation. Later, it has excellent performance in lung nodule detection and blood vessel extraction on the fundus retina. Including the CT image segmentation of liver tumor lesions. In specific implementation, this type of method can use deep features to locate liver tissue regions and use shallow features to achieve accurate segmentation results. Many medical image segmentation problems are improved based on U-Net. According to the adopted form of U-Net network architecture, it can be divided into single network liver tumor segmentation method, multi-network liver tumor segmentation method, and u. A liver tumor segmentation method combining Net network and traditional methods. Regardless of the calculation and memory performance, the 3D network can combine the image layer information to ensure a change continuity between the interlayer image masks, and the segmentation effect is better than 2D.

Considering clinical suitability and segmentation accuracy as well as processing time, our goal is to develop an efficient, robust, and accurate method for tumor segmentation. Therefore, in this paper, a deep learning method based on learning and decoding layered features with multiple spatial frequencies is proposed to achieve 3D liver tumor segmentation from CT images. The main contributions of this work are three-fold:

- Due to observe the CT liver tumor image can be decomposed to describe the structure of the smooth change (such as the shape of the tumor) mutations in the low spatial frequency components and describe the details of (the edge of the tumor, for example) the high spatial frequency components, so we use the octave convolution (28) encoder block for building characteristics, and use them to study neural network layered multiple frequency characteristics of multiple levels.
- We propose to decompose the convolution feature graph into two groups at different spatial frequencies and process them with different extended convolution at their corresponding frequencies (one octave apart). Storage and computation can be saved because the resolution of low frequency graphs can be reduced. This also helps each layer to have a larger receive field to capture more contextual information. Importantly, the proposed blocks are fast in practice and can reach speeds close to the theoretical limit.
- More importantly, we introduce deep supervision to the hidden layer, which can accelerate the optimization convergence speed and improve the prediction accuracy.
- In addition, the proposed network is superior to the benchmark U-Net in terms of segmentation performance and computing overhead, while achieving better or comparable performance to the latest approach on open data sets.

## 2. Methods

U-Net is modified on the basis of the existing CNN structure for classification, that is, the original fully connected layer of CNN is changed into a convolutional layer. FCN is composed of convolution and deconvolution. Through the process of convolution and deconvolution, based on end-to-end learning, the classification of each pixel of the image is completed, thereby realizing the segmentation of the entire input image. U-Net realizes the semantic segmentation of images through an end-to-end network structure. The end-to-end network can reduce manual preprocessing and subsequent processing and make the model from the original input to the final output as much as possible. The network learns the features by itself, and the extracted features are also integrated into the algorithm. The network model can be automatically adjusted according to the data, thereby increasing the overall fit of the model, and the cost of end-to-end network learning is lower than that of non-end-to-end network structure.

### 2.1. Encoder Part

**Figure 1:**
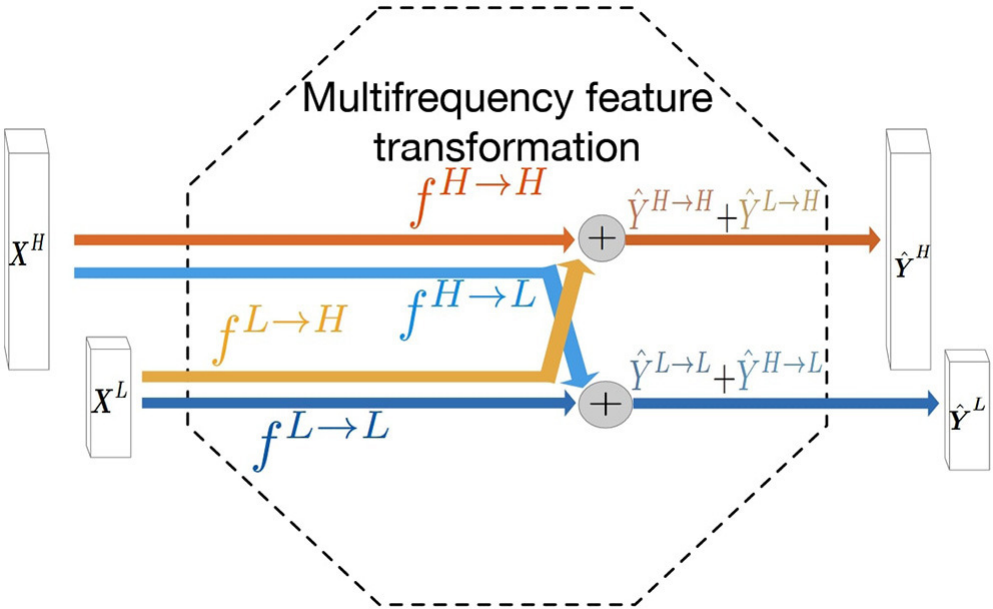
Computation graph of the multifrequency feature transformation of octave convolution. The operation mainly contains two processes of the inter-frequency information exchange (*f*^*L*→*H*^ and *f*^*H*→*L*^) and intra-frequency information update (*f*^*L*→*L*^ and *f*^*H*→*H*^).

The liver tumors often have varying sizes and shapes. The low- and high- frequency components of tumors focus on capturing the style of tumor and edge information, respectively. Motivated by this observation, we hypothesize that adopting a multi-frequency feature learning approach may be beneficial for segmenting the tumor from liver CT images. Therefore, the octave convolution (28) is adopted as an extractor for multifrequency features in this work. The computational graph for multifrequency feature transformations of the octave convolution is illustrated in Figure 1. Let *X*^*H*^ and *X*^*L*^ denote the inputs of high- and low- frequency feature maps, respectively. The high- and low-frequency outputs of the octave convolution are given by *Ŷ*^*H*^ = *f*^*H*→*H*^(*X*^*H*^) + *f*^*L*→*H*^(*X*^*L*^) and *Ŷ*^*L*^ = *f*^*L*→*L*^(*X*^*L*^) + *f*^*H*→*L*^(*X*^*H*^), where *f*^*H*→*H*^ and *f*^*L*→*L*^ denote two standard convolution operations for intra-frequency information update, whereas *f*^*H*→*L*^ and *f*^*L*→*H*^ denote the process of inter-frequency information exchange. Specifically, *f*^*H*→*L*^ is equivalent to first down-sampling the input by average-pooling with a scale of two and then applying a standard convolution for feature transformation, and *f*^*L*→*H*^ is equivalent to up-sampling the output of a standard convolution by nearest interpolation with a scale of two.

**Figure 2:**
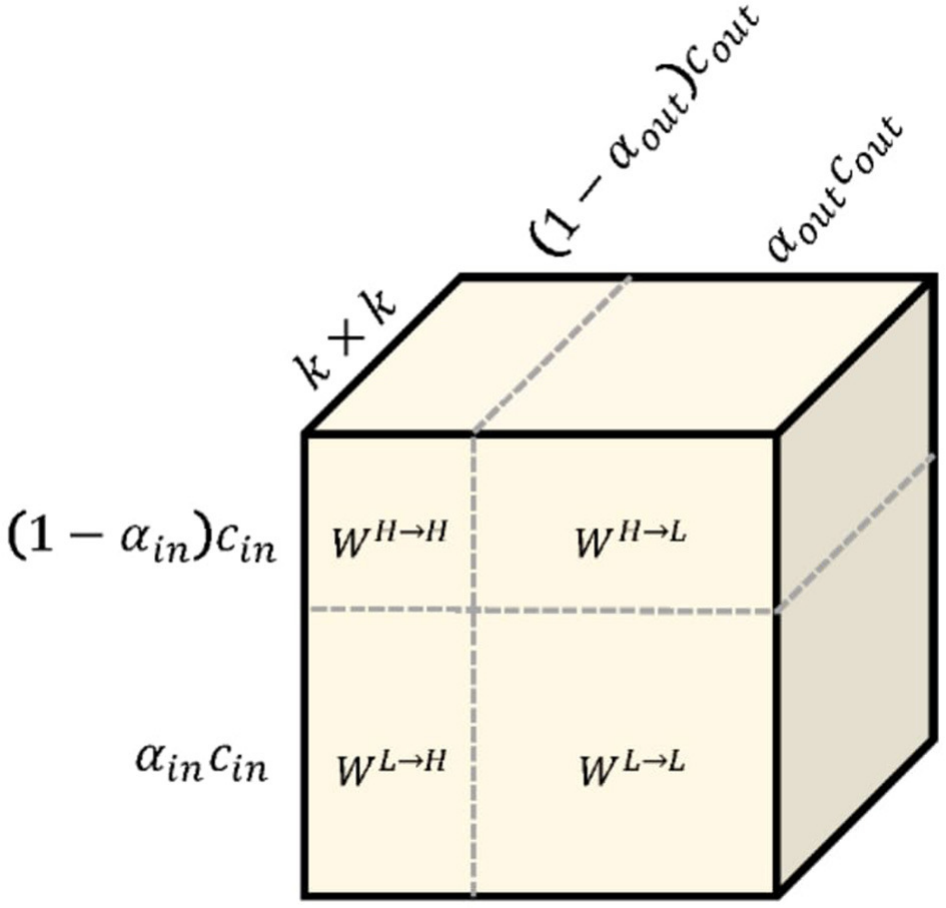
The octave convolution kernel. *k* × *k* octave convolution kernel *W* is equivalent to vanilla convolution kernel because they have exactly the same number of parameters.

To calculate these items, working (28) splits the convolution kernel *W*into two components *W* = [*W*^*H*^, *W*^*L*^]is responsible for convolved with *X*^*H*^and *X*^*L*^. Each component can be further divided into in-frequency and in-frequency parts: *W*^*H*^ = [*W*^*H*→*H*^, *W*^*L*→*H*^]and *W*^*L*^ = [*W*^*L*→*L*^, *W*^*H*→*L*^], whose parameter tensor shape is shown in Figure 2. Especially for the high-frequency feature graph, we use A to calculate its regular convolution for in-frequency update at the position (*p, q*)and for inter-frequency communication. We can fold the up-sampling of the feature tensor *X*^*L*^into convolution without explicit calculation and storage of the up-sampling function as follows:

(1) $$\begin{array}{l} Y_{p,q}^{H}=Y_{p,q}^{H\to H}+Y_{p,q}^{L\to H}\\ \;=\sum_{i,j\in N_{k}}W_{i+\frac{k-1}{2},j+\frac{k-1}{2}}^{H\to H}X_{p+i,q+j}^{H}\\ \;+\sum_{i,j\in N_{k}}W_{i+\frac{k-1}{2},j+\frac{k-1}{2}}^{L\to H}X_{\left(\left\lfloor \frac{p}{2}\right\rfloor +i\right),\left(\left\lfloor \frac{q}{2}\right\rfloor +j\right)}^{L} \end{array}$$

where ⌊·⌋represents a lower bound operation. Similarly, for low-frequency characteristic graphs, we use regular convolution to calculate in-frequency update. Note that since the graph is an octave lower, the convolution is also low frequency W.R.T. High frequency coordinate space. For inter-frequency communication, we can fold the subsample of the feature tensor *X*^*H*^into the convolution again, as shown below:

(2) $$\begin{array}{l} Y_{p,q}^{L}=Y_{p,q}^{L\to L}+Y_{p,q}^{H\to L}\\ \;=\sum_{i,j\in N_{k}}W_{i+\frac{k-1}{2},j+\frac{k-1}{2}}^{⊤}X_{p+i,q+j}^{L}\\ \;+\sum_{i,j\in N_{k}}W_{i+\frac{k-1}{2}}^{H\to L},j+\frac{k-1}{2}\;X_{\left(2*p+0.5+i),(2*q+0.5+j\right)}^{⊤} \end{array}$$

where multiplying a factor 2 to the locations (*p, q*) performs down-sampling, and further shifting the location by a half step is to ensure the down-sampled maps are well-aligned with the input.

### 2.2. Decoder Part

Deconvolution is a convolution operation, which is the inverse process of pooling. In U-Net, the pooling operation reduces the size of the input picture, but in the image segmentation process, each pixel needs to be classified, and finally a segmented image with the same dimension as the input picture is obtained. Therefore, the generated heat map (heat map) is restored to the original image dimensions. Through reverse training, deconvolution can achieve the effect of output reconstruction and input, so that the output image can be restored to the same dimension as the input image.

**Figure 3:**
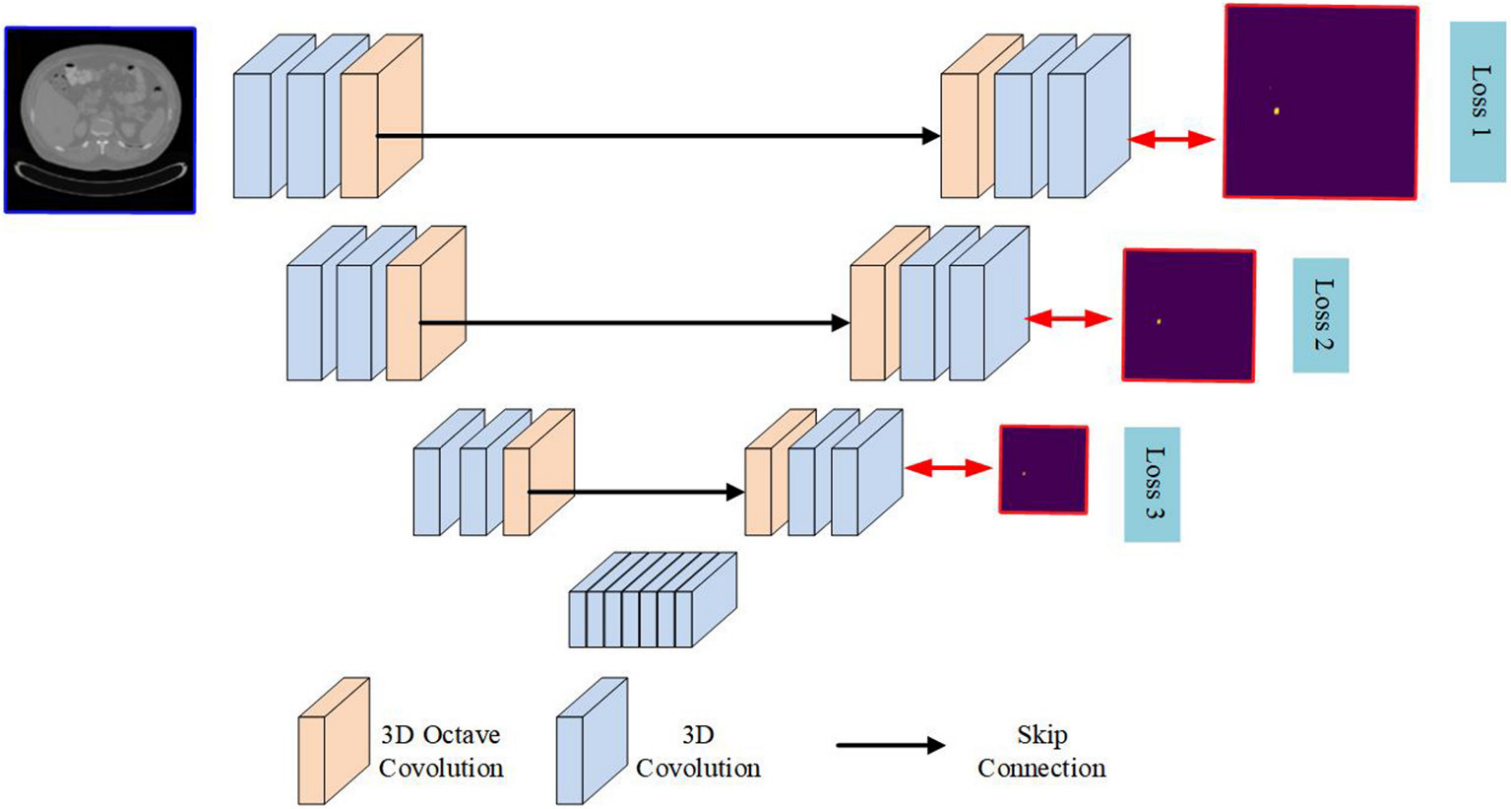
Detailed network architecture of the proposed network.

On the one hand, in the process of feature coding as shown in the Figure 3, although the spatial size of the feature graph gradually decreases, the feature graph gradually loses spatial details. This compression effect forces the kernel to learn more discriminations with higher levels of abstraction. On the other hand, multi-frequency feature extraction alone is not sufficient to perform dense pixel classification for liver tumor segmentation. A process is needed to decode the feature map to recover spatial detail and generate a high-resolution probabilistic map of the tumor. A simple way to do this is to use bilinear interpolation, which unfortunately lacks the ability to learn the decoding transformation that transpose convolution has. Therefore, we choose the transpose convolution to up-sample the feature.

### 2.3. The Proposed Network

**Figure 4:**
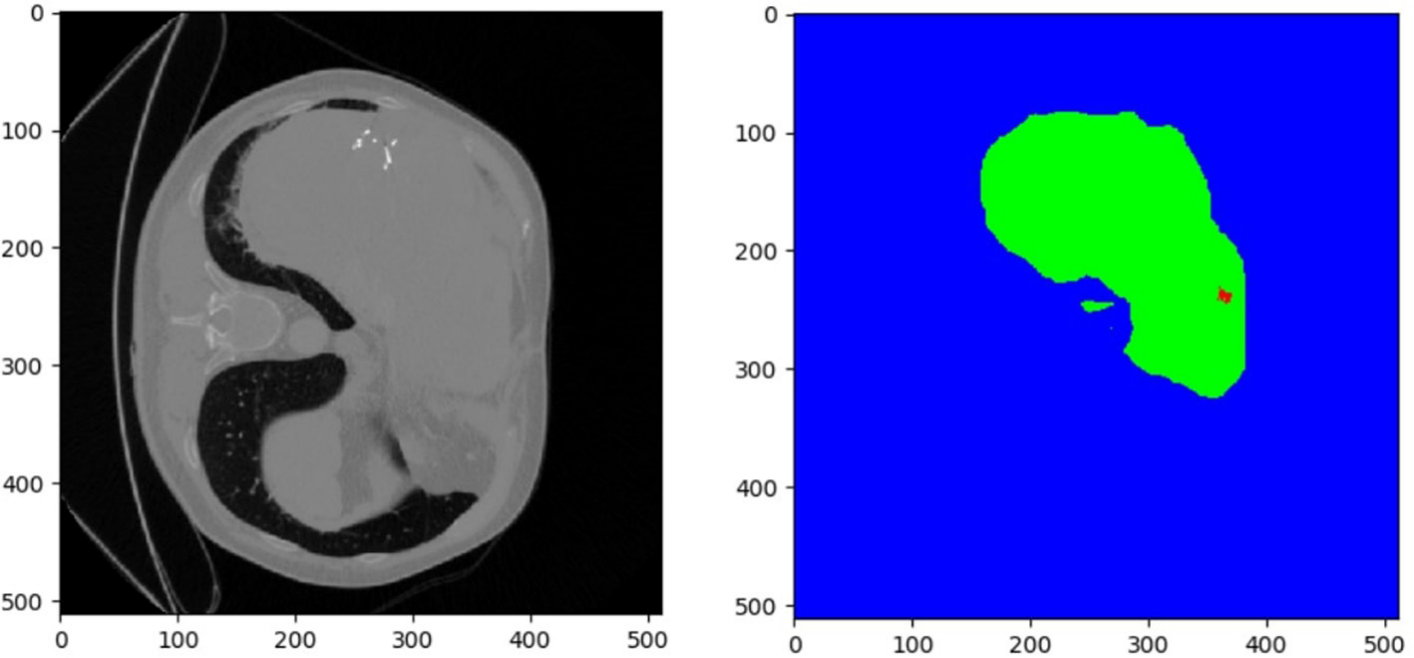
Training data provided by LiTS-challenge.

In this section, a novel encoder-decoder based neural network architecture called **OCunet** is proposed. After end-to-end training, the proposed OCunet is able to extract and decode layered multifrequency features for the segmentation of liver tumors in full-size CT images. The computational pipeline of OCunet consists of two main processes, namely feature encoding and decoding. By using octave convolution, we design multi-frequency feature encoder block and decoder block for hierarchical multi-frequency feature learning and decoding. By sequentially stacking multiple encoder blocks (as shown in Figure 4), layered multifrequency features can learn to capture details of the low frequency components that describe smooth changes in the structure (such as the main blood vessels) and the high frequency components that describe details of sudden changes (including the fine components), as shown in Figure 3.

### 2.4. Loss Function

The learning of the 3D network is formulated as a problem of minimizing the per-pixel binary classification error relative to the ground mask, but the optimization process is challenging. A major problem is the disappearance of the gradient, which makes the loss back propagation ineffective in the early layers. This problem is likely to be more serious in 3D and will inevitably slow down the convergence rate and the discriminating ability of the model. To address this challenge, we used additional monitoring injected into some hidden layers to counteract the negative effects of gradient disappearance. Specifically, we used an additional deconvolution layer to amplify some of the lower- and mid-level feature quantities, and then used the Softmax layer to obtain dense predictions for calculating classification errors. Using the gradient obtained from the prediction of these branches and the last output layer, the effect of gradient disappearance can be effectively mitigated.

Since the number of voxels belonging to the foreground is much smaller than the number belonging to the background (i.e., the liver), this problem of data imbalance usually leads to a prediction bias when using traditional loss functions. In order to solve this problem, the loss function, Dice coefficient (DICE), which represents the similarity measure between the ground truth and the predicted score graph, is proposed.

## 3. Experiments

### 3.1. Datasets

The LiTs dataset^1^ includes 130 contrast-enhanced 3D abdominal CT scan images from 6 different clinical sites, of which 130 cases are used for training and the remaining 70 are used for testing. The CT scan is accompanied by reference annotations of the liver and tumors made by a trained radiologist. The data set contains 908 lesions. The data set has significant differences in image quality, spatial resolution, and vision. The in-plane resolution is 0.6 × 0.6*mm*-1.0 × 1.0*mm*, slice thickness (layer spacing) is 0.45–6.0 mm, the axial slice size of all scans is fixed at 512 × 512 pixels, but the number of slices per scan It ranges from 42 to 1,026 sheets.

Further test data were provided by the Radiology Centre of the Medical University of Innsbruck. The data set contains CT scans of patients with liver cancer, with reference notes drawn up by medical scientists. Because deep learning methods can achieve better performance if the data has a consistent size or distribution, all data is normalized to strength values between [0,1] before starting optimization.

### 3.2. Implementation Details

Our OCunet was implemented with PyTorch library. We trained the network from scratch with weights initialized from Gaussian distribution. The learning rate was initialized as 0.1 and divided by 10 every 1,000 epochs. Each training epoch took around 2 min using a GPU of NVIDIA GTX 2080Ti.

### 3.3. Metrics

(1) Precision: Precision, or the positive predictive value, refers to the fraction of relevant instances among the total retrieved instances.
  (3) $$Precision=\frac{TP}{TP+FP}.$$
(2) Recall: Recall, also known as sensitivity, refers to the fraction of relevant instances retrieved over the total amount of relevant instances.
  (4) $$Recall=\frac{TP}{TP+FN}.$$
(3) Accuracy: Accuracy refers to the fraction of relevant instances among the total instances.
  (5) $$Accuracy=\frac{TP+TN}{TP+TN+FP+FN}.$$
(4) Specificity: Accuracy refers to the fraction of retrieved instances among the total amount of relevant instances.
  (6) $$Specificity=\frac{TN}{FP+TN}.$$
(5) DICE Score: also called the overlap index, is the most commonly used index to verify the segmentation of medical images, and it usually represents the repetition rate between the segmentation result and the mark. The value range of DICE is 0 1, 0 means real. The experimental segmentation result and the labeling result deviate seriously, and 1 means that the experimental segmentation result and the labeling result completely coincide. It is defined as follows:
  (7) $$Dic(A,B)=\frac{2|A\cap B|}{\left|A|+|B\right|}.$$

where *A* is the estimated maps, *B* denotes the ground truth, |*A* ∩ *B*| represents the number of pixels common to both images. The higher value of the dice coefficient denotes the better segmentation accuracy.

### 3.4. Evaluation on Test Data

**Figure 5:**
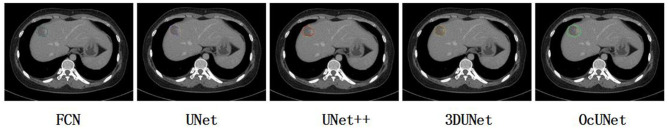
Example of tumor segmentation results from a testing image.

**Figure 6:**
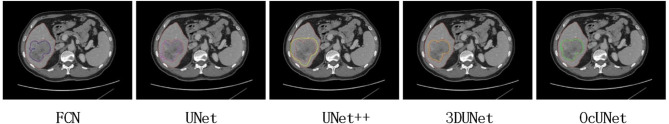
Compared results of tumor segmentation with different methods.

Figures 5, 6 show tumor segmentation results from training and test images, respectively. The red is a liver tumor. We compare the basic facts with the results generated by FCN, U-Net, UNet++, and 3DU-Net. In order to visualize the simplicity of the results caused by network differences, here we train the network only on the axial plane. In Figure 5, by FCN, U-Net, UNet++, and 3DU-Net provide results showed in the first, second, third, and fourth columns, we can see that in the FCN and U-Net segmentation results, residual connection can distinguish to some extent of tumor, but will miss part should belong to the tumor tissue. In UNet++ more accurate segmentation results can be predicted through intense connection, but compared with the result of a split, a split less than 3DU-Net still exists, thanks to combat training strategy, and can recognize more voxels belonging to the tumor. In the Figure 6, the results obtained from the test image show a similar appearance to the training image. However, it can be seen that liver tumors produced by 3DUNet are segmented more accurately. Although the segmentation results provided by 3DU-Net still have some unsegmented tumor tissue, it has been significantly improved compared to the other two methods, demonstrating the effectiveness of the algorithm. The quantitative results are reported in Table 1.

**Table 1:** Comparing different methods with the proposed dataset on the liver tumor segmentation task.

**Metrics**	**FCN**	**U-Net**	**UNet++**	**3DU-Net**	**3D Attention**	**OCunet**
Precision	0.872	0.896	0.901	0.914	0.926	0.939
Recall	0.923	0.930	0.931	0.925	0.951	0.962
Accuracy	0.912	0.930	0.942	0.951	0.956	0.959
Specificity	0.909	0.917	0.918	0.957	0.966	0.967
DICE	0.923	0.942	0.945	0.958	0.961	0.963

### 3.5. Ablation Study

In this section, we conduct experiments to investigate the effectiveness of different modules of our model. Starting from our baseline, we gradually inject our modifications on the whole structure. The results are summarized in Table 2, from which we can see that octave convolution is an effective block for liver tumor segmentation. In addition, we can find that the deep supervision can promote the performance the proposed method.

**Table 2:** Ablation study results.

**Metrics**	**Precision**	**Recall**	**Accuracy**	**Specificity**	**DICE**
w/o Octave Conv.	0.921	0.939	0.938	0.942	0.947
w Octave Conv.	0.928	0.946	0.944	0.950	0.951
Add 1 Loss	0.930	0.951	0.948	0.958	0.957
Add 2 Loss	0.936	0.959	0.952	0.962	0.960
OCunet	0.939	0.962	0.959	0.967	0.963

## 4. Conclusion

In this work, we propose a new network for segmentation of liver tumors. We solve the problem of reducing the extensive spatial redundancy in the original CNN model, and propose a novel Octave convolution operation to store and process the low frequency and high frequency features respectively to improve the model efficiency. In addition to octave convolution, the well-designed OCunet can also extract layered features with multiple spatial frequencies and reconstruct accurate tumor segmentation. Thanks to the design of layered multi-frequency features, OCunet is superior to the baseline model in terms of segmentation performance and computational overhead. A large number of experiments show that the proposed method based on octave convolution converges quickly and can produce high quality segmentation results.

At present, the development direction of deep learning in liver tumor segmentation is mainly concentrated in the following points: (1) The training of deep learning algorithms needs to rely on a large number of data sets, and due to its particularity and sensitivity, medical images need to be manually obtained and labeled by experts. The process is very time-consuming. Therefore, it is not only necessary for medical providers to provide more data support, but also to adopt enhanced methods for the data set to increase the size of the data set. The use of three-dimensional neural network and network deepening is a future research direction of this field; (2) The use of multi-modal liver images for segmentation and the combination of multiple different deep neural networks to extract deeper image information and improve the accuracy of liver tumor segmentation are also a major research direction in this field; (3) Currently most medical image segmentation uses supervised deep learning algorithms. However, for some rare diseases that lack a large amount of data support, supervised deep learning algorithms cannot exert their performance. To overcome the lack of data for the available problems, some researchers will transfer the supervised field to the semi-supervised or unsupervised field. For example, the GAN network is proposed. Combining the GAN network with other higher-performance networks, further research can be carried out in the future.

## Data Availability Statement

The original contributions generated for the study are included in the article/supplementary material, further inquiries can be directed to the corresponding author/s.

## Author Contributions

BW and JY: writing. LA, BY, and ZY: supervised. All authors contributed to the article and approved the submitted version.

## Conflict of Interest

JA was employed by company Beijing jingzhen Medical Technology Ltd. The remaining authors declare that the research was conducted in the absence of any commercial or financial relationships that could be construed as a potential conflict of interest.

## Footnotes

^1^https://competitions.codalab.org/competitions/17094
